# Genome features of common vetch ( *Vicia sativa* ) in natural habitats

**DOI:** 10.1002/pld3.352

**Published:** 2021-10-07

**Authors:** Kenta Shirasawa, Shunichi Kosugi, Kazuhiro Sasaki, Andrea Ghelfi, Koei Okazaki, Atsushi Toyoda, Hideki Hirakawa, Sachiko Isobe

**Affiliations:** ^1^ Kazusa DNA Research Institute Kisarazu Japan; ^2^ RIKEN Yokohama Japan; ^3^ Institute for Sustainable Agro‐ecosystem Services, Graduate School of Agricultural and Life Sciences The University of Tokyo Nishitokyo Japan; ^4^ Japan International Research Center for Agricultural Sciences Tsukuba Japan; ^5^ National Institute of Genetics Mishima Japan

**Keywords:** common vetch, ddRAD‐Seq, genome sequence, natural populations, nucleotide diversity, single nucleotide polymorphism

## Abstract

Wild plants are often tolerant to biotic and abiotic stresses in their natural environments, whereas domesticated plants such as crops frequently lack such resilience. This difference is thought to be due to the high levels of genome heterozygosity in wild plant populations and the low levels of heterozygosity in domesticated crop species. In this study, common vetch ( *Vicia sativa* ) was used as a model to examine this hypothesis. The common vetch genome (2n = 14) was estimated as 1.8 Gb in size. Genome sequencing produced a reference assembly that spanned 1.5 Gb, from which 31,146 genes were predicted. Using this sequence as a reference, 24,118 single nucleotide polymorphisms were discovered in 1243 plants from 12 natural common vetch populations in Japan. Common vetch genomes exhibited high heterozygosity at the population level, with lower levels of heterozygosity observed at specific genome regions. Such patterns of heterozygosity are thought to be essential for adaptation to different environments. The resources generated in this study will provide insights into de novo domestication of wild plants and agricultural enhancement.

## 1 INTRODUCTION

Wild plants, including weeds that have not yet been domesticated or cultivated, generally possess characteristics that allow them to survive and propagate in their natural environments when challenged by local biotic and abiotic stresses (Mammadov et al., 2018). The resilience exhibited by wild plants is thought to be due to their high levels of genetic heterogeneity (Cançado, 2011). Indeed, genetic heterogeneity was effective in suppressing disease when populations of genetically diversified crops were planted together in the same fields (Zhu et al., 2000).

In contrast with wild plants, crop plants have lost their natural survival traits as a result of the extremely low levels of genetic heterogeneity found in monoculture crop species (Mundt, 2002). Therefore, disease, insect, and weed controls are essential in commercial crop cultivation to reduce losses and maximize yields. This requires additional crop management costs for farmers, for example, for labor and agrochemicals. There are two main reasons for the low genetic heterogeneity in crop species. One reason is crop domestication (Izawa et al., 2009), in which only a few plants possessing desirable phenotypes, such as large fruit size, non‐seed shattering, and long‐seed dormancy, are selected from the broad genetic pools of wild plants. The second reason is selective breeding for desirable traits. While valuable for stabilizing crop phenotypes such as yield, these selective processes have reduced genetic diversity in monoculture crops by purging diverse germplasms (Fu, 2015). During domestication and selective breeding, small numbers of alleles that have large effects on phenotypic variations have often been targeted, further reducing the genetic diversity within cultivated varieties (Fernie & Yan, 2019).

While remaining more diverse than crop species, wild plant populations have also experienced loss of genetic heterogeneity at some loci, though in wild plants this is due to directional selection and genetic drift. For example, natural populations of Arabidopsis have lost genetic heterogeneity at flowering loci to synchronize flowering time (Mendez‐Vigo et al., 2011), which is beneficial for propagation under natural conditions. This suggests that genome‐wide genetic heterogeneity is not necessarily required for wild plant populations and that small numbers of loci could become fixed under certain selective conditions. This suggests that it would be possible to generate new plant populations with (a) fixed domestication loci with suitable alleles for agricultural traits and (b) high general levels of genetic diversity elsewhere in the genome. Such plant populations could be used as crop species, as proposed by Litrico and Violle (2015), and would possess natural resistance and suppression traits, as a result of high heterogeneity, that would enhance population resilience to biotic and abiotic stresses. As favorable agricultural alleles would be fixed, the benefits of genetic heterogeneity would exist alongside desirable agricultural traits. Mixtures of heterozygous plant populations have already been used as crops in allogamous species such as onion and clover. However, the potential benefits of genetic heterogeneity for autogamous plants such as legumes remain unclear.

Common vetch (*Vicia sativa*), a wild legume commonly found in open fields, was partially domesticated and cultivated in the past (Bryant & Hughes, 2011). Common vetch therefore has crop potential and can serve as a model for examination of genetic heterogeneity and domestication. The first step is to evaluate the levels of genetic heterogeneity in wild common vetch populations. However, no genome sequence data is available in *Vicia* including common vetch, while those of *Pisum sativum*, a member of Fabeae to which *Vicia* belongs, has been publicly available (Kreplak et al., 2019). Moreover, in common vetch, at least three different chromosome numbers (2n = 10, 12, and 14) have been reported (Ladizinsky, 1998; Ladizinsky & Waines, 1982). In this study, a reference sequence for common vetch was developed, and single nucleotide polymorphism (SNP) analysis with double‐digest restriction‐site associated DNA sequencing (ddRAD‐Seq) was used to evaluate heterogeneity in genomes of common vetch populations.

## 2 MATERIALS AND METHODS

### 2.1 Plant materials

**FIGURE 1:**
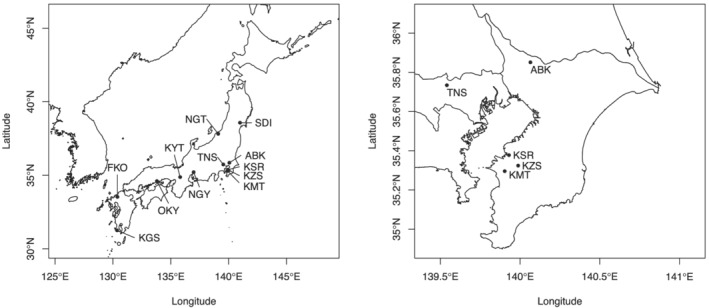
Sampling locations in Japan. Three‐letter codes indicate sampling locations in Japan: ABK: Abiko, Chiba; FKO: Fukuoka; KGS: Kagoshima; KMT: Kimitsu, Chiba; KSR: Kisarazu, Chiba; KYT: Kyoto; KZS: Kazusa, Chiba; NGT: Niigata; NGY: Nagoya, Aichi; OKY: Okayama; SDI: Sendai, Miyagi; and TNS: Tanashi, Tokyo

A standard inbred line of common vetch (*V. sativa*), KSR5, was established from a wild plant collected from Kisarazu, Chiba, Japan, by self‐pollination for more than three generations. KSR5 was used for genome and transcriptome sequencing analysis. For genetic diversity analysis, 1243 plants were collected from 12 locations across the latitude from 31.3°N to 38.8°N in Japan (Figure 1, Table S1). In addition, eight accessions from France, Germany, Greece, Iran, Italy, and Tunisia were obtained from the NIAS Genebank, Tsukuba, Japan (Table S1). Genomic DNA was extracted from young leaves with a DNeasy Plant Mini Kit (Qiagen, Hilden, Germany).

### 2.2 Chromosome observation

Root tips of 2‐day‐old seedlings of KSR5 were treated with 0.05% colchicine for 18 h, fixed with 1:3 acetate:ethanol for 2 h, and washed three times with water. Cell walls of the root tips were digested with 2% cellulase (SERVA Electrophoresis GmbH, Heidelberg, Germany), 2% macerozyme (SERVA Electrophoresis GmbH), and 0.1‐M sodium acetate for 4 h at 37°C. The root tip cells spread on a glass slide were fixed again with 1:3 acetate:ethanol and dried at room temperature. Chromosomes were stained with 1 μg/ml DAPI (4,6‐diamidino‐2‐phenylindole) in Fluoro‐KEEPER Antifade Reagent (Nacalai Tesque, Kyoto, Japan) and were observed under a confocal laser scanning microscope, LSM700 (Carl Zeiss, Oberkochen, Germany). Chromosome length was measured with ImageJ (Schneider et al., 2012).

### 2.3 Sequencing analysis of the common vetch genome

Genomic DNA from KSR5 was used to construct one paired‐end (insert size of 500 bp) and four mate‐pair sequencing libraries (insert sizes of 2, 5, 10, and 15 kb) in accordance with manufacturer protocols (Illumina, San Diego, CA, USA). Libraries were then sequenced using a HiSeq2000 instrument (Illumina). A long insert library for KSR5 was also prepared and sequenced on an RSII instrument (PacBio, Menlo Park, CA, USA). The paired‐end sequence reads were used for genomic size estimation based on *k*‐mer frequency (*k* = 17) using Jellyfish (Marcais & Kingsford, 2011). The paired‐end and mate‐pair reads were assembled and scaffolded with SOAPdenovo2 (Luo et al., 2012). Gaps, represented by Ns in the scaffold sequences, were filled by PBjelly (English et al., 2012) with PacBio reads, in which sequence errors were corrected with the paired‐end reads by proovread (Hackl et al., 2014). Contaminated sequences were removed by BLASTN search (Altschul et al., 1990), with an E‐value cutoff of 1E−10 and length coverage of ≥10%, against sequences from potential contaminating resources such as organelles (the plastid and mitochondrion genome sequences of *Lotus japonicus* and *Vicia faba*: KF042344, AP002983, JN872551, and KC189947), bacteria and fungi (NCBI bacteria and fungi), human (hg19), and artificial sequences (UniVec and PhiX). The resulting sequences that were ≥1000 bp in size were selected and designated VSA_r1.0 as a draft common vetch genome. Completeness of the assembly was assessed with sets of a Benchmarking Universal Single‐Copy Orthologs (BUSCO) (Simao et al., 2015).

### 2.4 RNA sequencing and assembly

Total RNA was extracted from 10 tissue samples (roots, seedlings, stems, apical buds, immature and mature leaves, tendrils, flower buds, flowers, and pods) using an RNeasy Mini Kit (Qiagen) and treated with RQ1 RNase‐Free DNase (Promega, Madison, WI, USA) to remove contaminating genomic DNA. RNA libraries were constructed in accordance with the TruSeq Stranded mRNA Sample Preparation Guide (Illumina). Nucleotide sequences were obtained with a MiSeq instrument (Illumina) in the paired‐end 301 bp mode. Low‐quality reads were removed using PRINSEQ (Schmieder & Edwards, 2011), and adapter sequences were trimmed with fastx_clipper (parameter, ‐a AGATCGGAAGAGC) in the FASTX‐Toolkit (http://hannonlab.cshl.edu/fastx_toolkit). The resulting reads were assembled using Trinity (Grabherr et al., 2011) with parameters of –min_contig_length 100, –group_pairs_distance 400, and –SS_lib_type RF to generate a non‐redundant gene sequence set.

### 2.5 Repetitive sequence and RNA coding gene analysis

A de novo repeat sequence database for VSA_r1.0 was built using RepeatScout (Price et al., 2005) (version 1.0.5). Repetitive sequences in VSA_r1.0 were searched for using RepeatMasker (version 4.0.3) (http://www.repeatmasker.org) based on known repetitive sequences registered in Repbase (Bao et al., 2015) and the de novo repeat libraries. Transfer RNA genes were predicted using tRNAscan‐SE (version 1.23) (Chan & Lowe, 2019) with the default parameters, and ribosomal RNA (rRNA) genes were predicted using BLASTN searches with an E‐value cutoff of 1E−10, with the *Arabidopsis thaliana* 18S rRNA (accession number: X16077) and 5.8S and 25S rRNAs (accession number: X52320) used as query sequences.

### 2.6 Protein‐coding gene prediction and annotation

Putative protein‐coding genes in VSA_r1.0 were identified with a MAKER pipeline (version 2.31.8) (Cantarel et al., 2008) including ab initio‐, evidence‐, and homology‐based gene prediction methods. For this prediction, the non‐redundant gene sequence set generated from the RNA‐Seq analysis and peptide sequences predicted in the genomes of four Fabaceae members, namely, *Arachis duranensis* (V14167.a1.M1) (Bertioli et al., 2016), *L. japonicus* (rel. 3.0) (Sato et al., 2008), *Medicago truncatula* (4.0v1) (Young et al., 2011), and *Phaseolus vulgaris* (v1.0) (Schmutz et al., 2014), were used as a training data set. In addition, BRAKER1 (version 1.3) (Hoff et al., 2016) was used to complete the gene set for VSA_r1.0. To select high‐confidence genes, transposable elements (TEs) were removed using BLASTP searches against the NCBI non‐redundant (nr) protein database with an E‐value cutoff of 1E−10 and by using InterProScan (version 4.8) (Jones et al., 2014) searches against the InterPro database with an E‐value cutoff of 1.0.

The contig sequences of common vetch (VSA_r1.0) were compared with those of *P. sativum* (Pisum_sativum_v1a) (Kreplak et al., 2019) with D‐GENIES (Cabanettes & Klopp, 2018). Putative VSA_r1.0 genes were clustered using CD‐hit (version 4.6.1) (Li & Godzik, 2006) with the UniGene set of the four Fabaceae members as above with the parameters c = 0.6 and aL = 0.4. The predicted genes were annotated with plant gene ontology (GO) slim categories and euKaryotic clusters of Orthologous Groups (KOG) categories (Tatusov et al., 2003), and mapped onto the Kyoto Encyclopedia of Genes and Genomes (KEGG) reference pathways (Ogata et al., 1999).

Gene expression was quantified by mapping the RNA‐Seq reads onto VSA_r1.0 using HISAT2 (Kim et al., 2015) to quantify the RNA transcription followed by normalization to determine fragments per kilobase of exon per million mapped fragments (FPKM) values using StringTie (Pertea et al., 2015) and Ballgown (Frazee et al., 2015) in accordance with the published protocol (Pertea et al., 2016).

### 2.7 Genetic diversity analysis

Genome‐wide sequence variations in wild vetch populations were analyzed by a double‐digest restriction‐site associated DNA sequencing (ddRAD‐Seq) technique (Peterson et al., 2012). In accordance with the workflow established in our previous study (Shirasawa et al., 2016), genomic DNA samples from each line were digested with the restriction enzymes *Pst*I and *Eco*RI to prepare ddRAD‐Seq libraries, which were then sequenced on a HiSeq2000 (Illumina) instrument in paired‐end 93 bp mode. Low‐quality sequences were removed and adapters were trimmed using PRINSEQ (Schmieder & Edwards, 2011) and fastx_clipper in the FASTX‐Toolkit (http://hannonlab.cshl.edu/fastx_toolkit), respectively. The remaining high‐quality reads were mapped onto VSA_r1.0 as a reference using Bowtie2 (Langmead & Salzberg, 2012). The resultant sequence alignment‐map format (SAM) files were converted to binary sequence alignment‐map format (BAM) files and subjected to SNP calling using the mpileup option of SAMtools (Li et al., 2009) and the view option of BCFtools. High‐confidence SNPs were selected using VCFtools (Danecek et al., 2011) with the following criteria: (1) depth of coverage ≥5 for each line, (2) SNP quality scores of 999 for each locus, (3) minor allele frequency ≥0.05 for each locus, and (4) proportion of missing data <0.5 for each locus. The effects of SNPs on gene function were predicted using SnpEff v4.2 (Cingolani et al., 2012).

Fixation indexes (Fst), nucleotide divergency (π) values, and heterozygosity levels for SNP sites of each population were calculated using the weir‐fst‐pop, site‐pi, and het options in VCFtools (Danecek et al., 2011), respectively. Principal component analysis (PCA) was performed to determine the relationships among samples using TASSEL (Bradbury et al., 2007) and population structure was investigated using ADMIXTURE (Alexander et al., 2009). The R package WGCNA (Langfelder & Horvath, 2008) was used for SNP module detection.

## 3 RESULTS

### 3.1 Chromosome number of a common vetch line, KSR5

**FIGURE 2:**
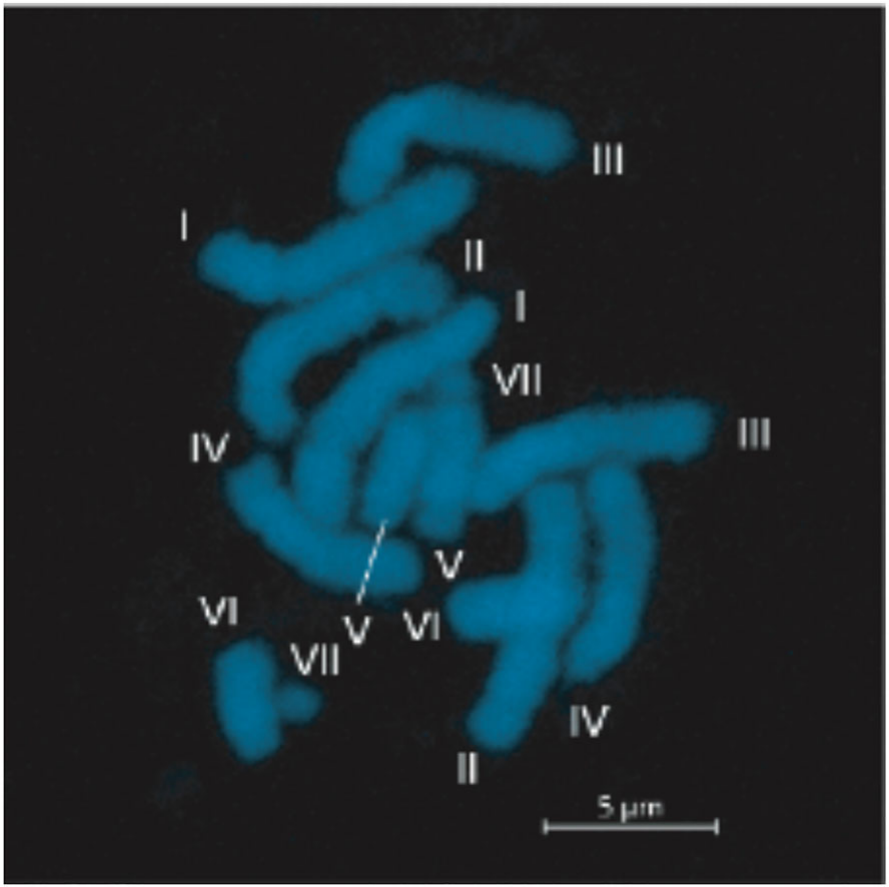
Chromosomes of the common vetch KSR5. Roman numerals indicate chromosome pairs, which order is based on chromosome length (I to VII). Bar = 5 μm

A total of 14 chromosomes, including two mini chromosomes, were observed in metaphase cells of root tips of the standard inbred line, KSR5 (Figure 2, Table 1). Relative length of the chromosomes was measured in five cells and sorted by the length order. In accordance with the chromosome length, the 14 chromosomes were grouped into seven pairs (I to VII), suggesting that the genome of KSR5 was 2n = 14. The relative length of the longest chromosome (I) was 22.3% of the total length of haploid genome, followed by 21.0% (II), 18.6% (III), 16.1% (IV), 10.3% (V), 9.1% (VI), and 2.7% (VII).

**TABLE 1:** Relative chromosome length of *Vicia sativa* , KSR5

Chromosome	Relative length (%)	S.D.^a^
I	22.3	0.7
II	21.0	0.7
III	18.6	1.3
IV	16.1	1.6
V	10.3	0.7
VI	9.1	0.6
VII	2.7	1.0

^a^ Standard deviation (*n* = 10).

### 3.2 Sequencing and genome assembly

**FIGURE 3:**
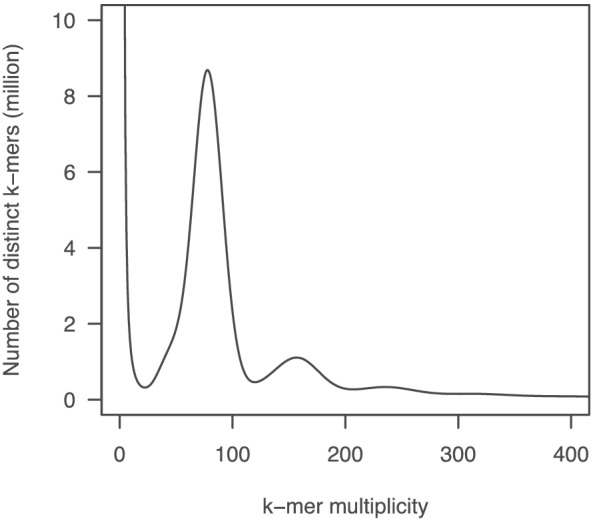
Genome size estimation for *Vicia sativa* with the distribution of the number of distinct *k*‐mers (*k* = 17) with the given multiplicity values

The standard inbred line of common vetch (*V. sativa*), KSR5, was sequenced. In total, 1.8 billion paired‐end reads corresponding to 186.7 Gb (Table S2) were obtained. The distribution of distinct *k*‐mers (*k* = 17) showed a single main peak at multiplicities of 78 with minor peaks (Figure 3). The size of the common vetch genome was estimated to be 1769 Mb. The paired‐end reads (105 × genome coverage) were assembled with mate‐pair reads of four libraries (146× genome coverage in total) to obtain 6487 thousand (k) scaffold sequences of total length 2.5 Gb with an N50 of 30.5 kb. After removing 6,421 k contaminated sequences and short scaffolds (<1 kb), sequence gaps presented by Ns in the remaining sequences were filled with PacBio long reads (3× genome coverage) to obtain a draft sequence of the common vetch genome, namely, VSA_r1.0. The total length of VSA_r1.0 was 1541 Mb and consisted of 54,083 sequences with an N50 of 90.1 kb (Table 2). Although 513 k gaps occupied 501 Mb in total (32.5%), the gene space was well represented in accordance with BUSCO examination, indicating 94.1% ortholog completion.

**TABLE 2:** Assembly statistics of the common vetch (*Vicia sativia)* genome assembly VSA_r1.0

	VSA_r1.0
Number of scaffolds	54,083
Assembly size (bp)	1,541,180,487
Scaffold N50 (bp)	90,105
Maximal scaffold (bp)	871,438
Number of gaps	513,235
Gap size (bp)	501,483,283
Complete and single‐copy BUSCO	77.5%
Complete and duplicated BUSCO	16.6%
Fragmented BUSCO	2.9%
Missing BUSCO	2.9%
Number of genes predicted	31,146

### 3.3 Repeat sequence analysis

Sequences totaling 782 Mb (51.9%) were identified as repeat elements such as transposons and retrotransposons (Table 3). Of this, sequences totaling 267 Mb were repeat sequences reported in other organisms, and sequences in the remaining 531 Mb were uniquely identified in VSA_r1.0. Of the previously reported repeats, long terminal repeat retroelements were predominant (200 Mb). Furthermore, 109,151 simple‐sequence repeats with 52,874 di‐, 39,198 tri‐, 12,354 tetra‐, 3414 penta‐, and 1311 hexa‐nucleotide repeat motifs were also found.

**TABLE 3:** Repeat sequences in the VSA_r1.0 assembly

Repeat type	Length occupied (bp)	%
SINEs^a^	85,029	0.0
LINEs^a^	10,462,622	0.7
LTR elements^a^	200,723,246	13.0
DNA elements	15,595,575	1.0
Helitrons	1,469,970	0.1
Satellites	17,496,670	1.1
Simple repeats	17,496,670	1.1
Low complexity	4,468,370	0.3
Novel repeats	531,016,543	34.5
**Total** ^b^	**782,834,201**	**50.8**

^a^ SINEs: short interspersed nuclear elements; LINEs: long interspersed nuclear elements; and LTR: long terminal repeat.

^b^ Non‐redundant sequence length of the repeats overlapping in the genome.

### 3.4 Gene prediction and annotation

In total, 31,146 protein‐encoding genes, with average length of 1008 bp and N50 of 1419 bp, were predicted in VSA_r1.0 (Table 2). For the evidence‐based MAKER pipeline, 166 million (M) RNA reads from 10 tissue samples (Table S2) were assembled into 181,211 transcribed sequences and used to predict 27,880 genes (genes with .mk suffix). A further 3266 genes were predicted using an ab initio‐based method (genes with .br suffix). GO classification assigned 8878, 4059, and 13,752 genes to the GO slim terms of biological process, cellular component, and molecular function, respectively (Table S3). KOG analysis revealed 2766, 4888, and 4424 genes with significant similarities to genes involved in information storage and processing, cellular processing and signaling, and metabolism, respectively (Table S4). Finally, 1720 genes were mapped to KEGG metabolic pathways (Table S5).

**FIGURE 4:**
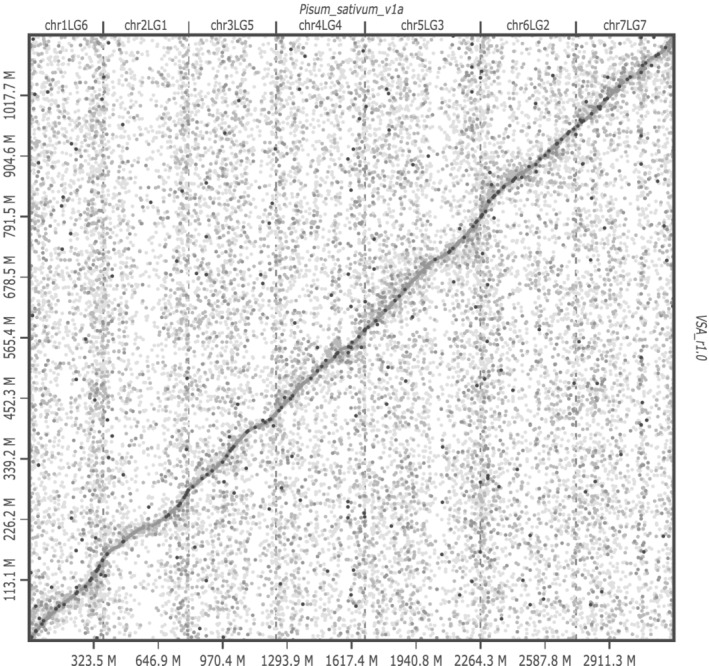
Comparative genome analysis of *Vicia sativa* and *Pisum sativum* . Dots indicate sequence similarity of the *V. sativa* genome (VSA_r1.0) on the vertical axis versus those of *P. sativum* (Pisum_sativum_v1a) on the horizontal axis

**FIGURE 5:**
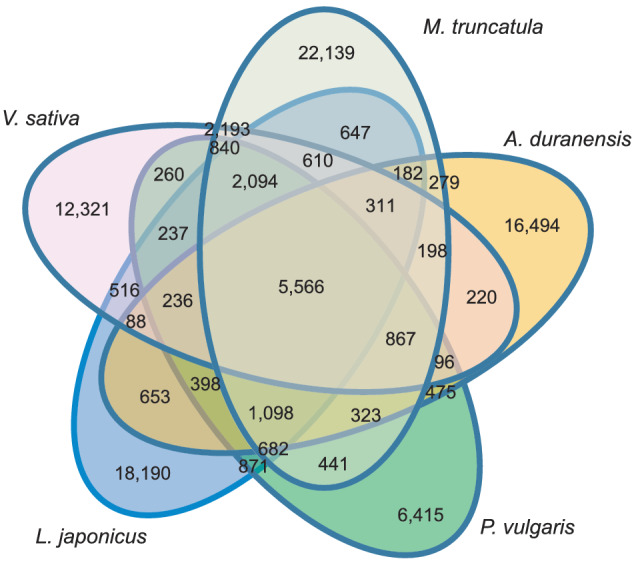
Venn diagram showing numbers of gene clusters in *Vicia sativa* and four additional Fabaceae species

A total of 137.9 Mb sequences of VSA_r1.0 were aligned on 121.7 Mb sequences across the *P. sativum* genome (Figure 4). Of the 137.9 Mb sequences, 42.4 Mb sequences were included within the gene regions. Gene sequences were highly conserved rather than intergenic regions among the members of Fabeae. Gene clustering analysis revealed 5566 gene clusters that were common to the five legume species tested (*V. sativa*, *A. duranensis*, *L. japonicus*, *M. truncatula*, and *P. vulgaris*) and 12,321 clusters that were unique to common vetch (Figure 5). In addition to mRNA sequences, 58 rRNA‐ and 1437 tRNA‐encoding genes were predicted.

### 3.5 Single nucleotide polymorphisms in natural populations

Genome‐wide SNPs were identified across the 12 common vetch populations from Japan, consisting of 1243 lines, and eight lines from France, Germany, Greece, Iran, Italy, and Tunisia from the NARO GeneBank (Tsukuba, Japan) (Table S1). Approximately 1.1 million ddRAD‐Seq reads per sample were obtained (Table S2) and 84.4% of the reads aligned to the VSA_r1.0 reference sequence. The ddRAD‐Seq reads covered 2.4 Mb (0.16%) of the reference assembly with ≥5 reads. Sequence alignments detected 46,715 high‐confidence SNPs (30.9% transitions and 69.1% transversions). SNP density was calculated as 1 SNP per 51 bp. When only the 12 populations from Japan were considered, the number of SNPs decreased to 24,118 (1 SNP per 100 bp), ranging from 4709 SNPs in the SDI population (1 SNP per 510 bp) to 10,040 SNPs in the ABK population (1 SNP per 239 bp) (Table 4).

**TABLE 4:** Cluster, heterozygosity, and nucleotide diversity calculated from SNPs of 12 common vetch natural populations in Japan

Population	Sampling location^a^	Number of individuals	Number of SNPs	Number of clusters (K)	Expected heterozygosity (He)	Observed heterozygosity (Ho)	Nucleotide divergency (π)
ABK	Abiko, Chiba, Japan	102	10,040	4	0.313	0.189	0.314
FKO	Fukuoka, Japan	97	9,795	7	0.318	0.057	0.319
KGS	Kagoshima, Japan	109	5,189	8	0.330	0.106	0.330
KMT	Kimitsu, Chiba, Japan	95	7,256	9	0.336	0.087	0.336
KSR	Kisarazu, Chiba, Japan	88	6,450	4	0.340	0.111	0.340
KYT	Kyoto, Japan	104	8,974	8	0.339	0.114	0.338
KZS	Kazusa, Chiba, Japan	97	7,243	4	0.334	0.147	0.334
NGT	Niigata, Japan	100	6,658	3	0.247	0.085	0.248
NGY	Nagoya, Aichi, Japan	102	6,891	5	0.335	.140	0.335
OKY	Okayama, Japan	99	9,649	11	0.337	0.085	0.336
SDI	Sendai, Miyagi, Japan	100	4,709	2	0.264	0.161	0.262
TNS	Tanashi, Tokyo, Japan	150	7,939	10	0.326	0.153	0.325

^a^ Geographical positions are indicated in Figure 1 and Table S1.

**FIGURE 6:**
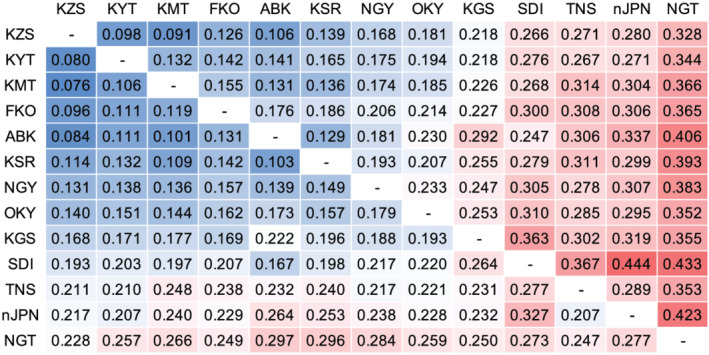
Heatmap of Fst between any combination of the 12 natural populations of *Vicia sativa* from Japan and one non‐Japanese groups. Upper and lower triangles indicate weighted and mean Weir and Cockerham Fst estimated. The colors in each block represent a continuum of Fst: low‐to‐high Fst are represented by blue to red via white. Three‐letter codes indicate sampling locations in Japan as indicated in the Figure 1, while nJPN indicates non‐Japanese group including eight lines from Greece, Italy, Germany, France, Tunisia, and Iran listed in the Table S1

**FIGURE 7:**
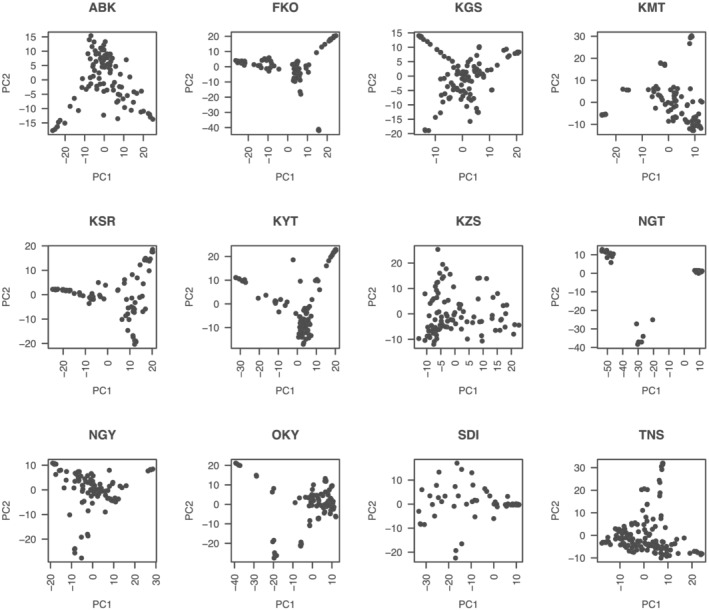
Principal component analysis of 12 natural populations of *Vicia sativa* from Japan

**FIGURE 8:**
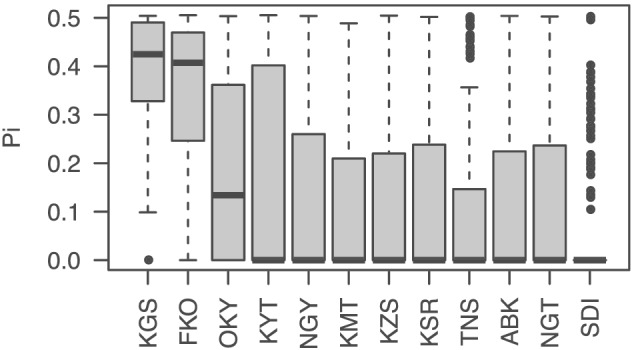
Nucleotide diversity (π) of the SNP module “cyan” (n = 190) across 12 natural populations of *Vicia sativa* in Japan. Three‐letter codes indicate sampling locations in Japan as indicated in the Figure 1

Fst values between any combinations of the 12 Japanese and one non‐Japanese populations were calculated with the SNPs. Whereas the Fst of nine populations were low, those of four populations, NGT, SDI, TNS, and non‐Japanese, were relatively high (Figure 6). PCA and admixture analysis indicated that there were 2–11 subpopulations in each of the 12 populations from Japan (Figure 7, Table 4, Figure S1). The observed heterozygosity scores were lower than the expected values, especially in FKO followed by NGT, OKY, and KMT (Table 4). Nucleotide divergency scores (π) at SNP sites were similarly distributed across 10 of the populations from Japan, with median values of 0.31–0.34. The remaining two populations, NGT and SDI, exhibited median values of ~0.25 (Table 4). Of the 46,715 high‐confidence SNPs, 24,118 clustered according to their π scores to generate 82 modules (Figure S2). Of these, the π scores of one cluster, “cyan,” which contained 190 SNPs, negatively correlated with the latitude of sampling location (Figures 1 and 8, Table S1). In total, 88 genes were associated with the 190 SNPs, and one of the genes (Vsa_sc30698.1_g030.1.mk) showed sequence similarity to the Arabidopsis gene for a MADS‐box protein, SUPPRESSOR OF OVEREXPRESSION OF CONSTANS1 (SOC1), known to be involved in the flowering pathway in plants. RNA expression analysis, in which 76.5% of RNA‐Seq reads were mapped on VSA_r1.0 as a reference, indicated that Vsa_sc30698.1_g030.1.mk was predominantly transcribed in tendrils (FPKM = 5.0) followed by apical buds (0.5) and stems (0.4). No expression was observed in the other seven tissues, that is, roots, seedlings, immature and mature leaves, flower buds, flowers, and pods.

## 4 DISCUSSION

We present a draft common vetch (*V. sativa*) genome sequence spanning 87.1% of the estimated size, 1769 Mb (Table 2, Figure 3), which size is closed to that estimated using flow cytometry (Macas et al., 2015). Although several legume genome sequences were released previously (Bauchet et al., 2019), this is the first report of a genome from the genus *Vicia*, which contains several agronomically important legume crops such as fava bean (*V. faba*). *Vicia* genomes are large (e.g., 1.8 Gb for *V. sativa* and 13 Gb for *V. faba*) due to their massive repetitive sequences, including TEs (Bryant & Hughes, 2011; Hill et al., 2005; Nouzova et al., 2001; Pearce et al., 1996), hampering de novo genome assembly in this genus (Bauchet et al., 2019). As might therefore be expected, more than half of the *V. sativa* genome assembly was composed of repetitive sequences (Table 3). The assembly contained up to 54,083 contig sequences and included 513 k gaps occupying >500 Mb (Table 2). The short‐read technology employed for sequencing might therefore be insufficient to span the repeats. Although construction of contiguous sequences from the short reads was challenging, a near complete gene set was successfully identified in the assembly (Table 2). Even though the fragmented genome sequences of *V. sativa* were aligned across the *P. sativum* genome (Figure 4), clustering analysis of the gene sequences would provide insights into the gene homoeology in legume species (Figure 5). The genome resources developed in this study will be invaluable for forthcoming gene discovery studies, such as transcriptome analysis and allele mining, in *Vicia*.

We reproducibly observed seven pairs of chromosomes (I to VII) in the root‐tip cells of KSR5 (Figure 2), among of which one pair (VII) was so small occupying only 2.7% of the total length of the seven chromosome pairs (Table 1). One type of mini chromosomes, so called B chromosomes which are comprised of repetitive sequence, have been reported in numerous groups of plants so far, but the biological function has not been known (Houben, 2017). B chromosomes are not necessary for the growth and normal development of organisms and show non‐Mendelian inheritance patterns (Houben, 2017). This could be one of the reasons for the different chromosome numbers in *V. sativa* (Ladizinsky, 1998; Ladizinsky & Waines, 1982; Navratilova et al., 2003). Further chromosome observations and fluorescence in situ hybridization with the repetitive sequences as probes across multiple lines would characterize and identify the mini chromosomes observed in this study. Alternatively, sterility of F1 hybrids derived from crosses between plants with different chromosome numbers should be analyzed to gain insights into the function of the small chromosomes. We indeed observed complete hybrid sterility in an F1 plant derived from a cross between KSR5 and the French line, IC403 (data not shown).

Twelve common vetch populations from Japan were examined, each of which contained 2–11 subpopulations (Figure 7, Table 4, Figure S1). This suggested that the numbers of founder plants were limited even in populations grown under natural environmental conditions. In accordance with the low Fst, Number of clusters (K), and nucleotide diversity (π) (Figure 6 and Table 4), it was suggested that small numbers of immigrants might contribute to establish NGT and SDI populations. Heterozygosity is thought to contribute strongly to the survival of plant populations under natural conditions (Cançado, 2011). Here, the observed heterozygosity was lower than expected (Table 4), indicating that heterozygosity in common vetch populations was high at the population level but low at the individual level due to self‐pollination. Such heterozygosity is reported in *V. faba* (El‐Rodeny et al., 2014; Link et al., 1994), suggesting that high heterozygosity at the population level might be sufficient to allow adaptation and survival under filed conditions in wild plants as well as cultivated species. Further studies based on ecology and evolution as well as genomics would be required to clarify the adaptation of the populations with different genetic parameters, for example, Fst, Number of clusters (K), nucleotide diversity (π), and heterozygosity.

Human domestication of wild plant species for agriculture involved selection of individual plants with desirable traits (Izawa et al., 2009; Vaughan et al., 2007). More recently, elite cultivars have been developed with enhanced yield performance to satisfy global food requirements (Hickey et al., 2019). The successive selection of small numbers of individual plants during these processes produced severe bottleneck effects and resulted in decreased genetic diversity and lower tolerance to biotic and abiotic stresses (Cançado, 2011). Heterozygosity at specific genome regions was also lost in some wild plants (Figure 8), as reported previously (Mendez‐Vigo et al., 2011). This suggested that genome‐wide genetic heterogeneity is not necessarily required for plants to survive under natural conditions. Recent studies have proposed de novo‐, super‐, or neo‐domestication (Fernie & Yan, 2019; Hickey et al., 2019; Vaughan et al., 2007), whereby genetic loci for agronomically important traits are introduced to cultivated crop varieties from wild plants. However, the high genetic heterozygosity levels from the wild donor plants should be retained during the development of new crops to avoid the bottleneck effects sustained during historic domestication of crop varieties (Litrico & Violle, 2015). Therefore, we propose that new domestication of wild plants should retain high heterozygosity at the population level to capitalize on beneficial traits that increase tolerance to abiotic and biotic stresses, but that agronomically important genetic loci should be fixed to maximize crop potential. The resources generated in this study will provide insights into the de novo domestication of wild plants to develop enhanced crop varieties.

## CONFLICT OF INTEREST

The Authors did not report any conflict of interest.

## AUTHOR CONTRIBUTIONS

K. Shirasawa conceived and coordinated the project; K. Shirasawa and K. Sasaki managed the plant materials; K. Shirasawa, K. Sasaki, K.O., A.T., and S.I. performed experiments and collected data; K. Shirasawa, S.K., A.G., K.O., and H.H. analyzed and interpreted the data; K. Shirasawa wrote the manuscript; and all authors read and approved the final manuscript.

## Supporting information


**Table S1** Plant materials.
**Table S2** Genome and transcriptome data.
**Table S3** Number of GO terms for protein‐encoding genes.
**Table S4** Number of KOG functions for protein‐encoding genes.
**Table S5** Number of genes mapped to KEGG pathways.

**Figure S1** Cross‐validation errors for 12 natural populations of *Vicia sativa* from Japan in admixture analysis.
Figure S2 Nucleotide diversity of SNP modules across 12 natural populations of *V. sativa* from Japan.

## Data Availability

Sequence data are available from the Sequence Read Archive (DRA) of DNA Data Bank of Japan (DDBJ) under accession numbers DRA004347 for whole genome sequencing, DRA004313 for RNA‐Seq, and DRA004301‐DRA004312 for ddRAD‐Seq (Table S2). The DDBJ accession numbers of the assembled sequences are BLWO01000001‐BLWO01054083. Genome information is available at Plant GARDEN (https://plantgarden.jp).

## References

[pld3352-bib-0001] Alexander, D. H. , Novembre, J. , & Lange, K. (2009). Fast model‐based estimation of ancestry in unrelated individuals. Genome Research, 19, 1655–1664. 10.1101/gr.094052.109 19648217 PMC2752134

[pld3352-bib-0002] Altschul, S. F. , Gish, W. , Miller, W. , Myers, E. W. , & Lipman, D. J. (1990). Basic local alignment search tool. Journal of Molecular Biology, 215, 403–410. 10.1016/S0022-2836(05)80360-2 2231712

[pld3352-bib-0003] Bao, W. , Kojima, K. K. , & Kohany, O. (2015). Repbase Update, a database of repetitive elements in eukaryotic genomes. Mobile DNA, 6, 11. 10.1186/s13100-015-0041-9 26045719 PMC4455052

[pld3352-bib-0004] Bauchet, G. J. , Bett, K. E. , Cameron, C. T. , Campbell, J. D. , Cannon, E. K. S. , Cannon, S. B. , Carlson, J. W. , Chan, A. , Cleary, A. , Close, T. J. , Cook, D. R. , Cooksey, A. M. , Coyne, C. , Dash, S. , Dickstein, R. , Farmer, A. D. , Fernández‐Baca, D. , Hokin, S. , Jones, E. S. , … Zhao, P. X. (2019). The future of legume genetic data resources: Challenges, opportunities, and priorities. Legume Science, 1(1), e16. 10.1002/leg3.16

[pld3352-bib-0005] Bertioli, D. J. , Cannon, S. B. , Froenicke, L. , Huang, G. , Farmer, A. D. , Cannon, E. K. , Liu, X. , Gao, D. , Clevenger, J. , Dash, S. , Ren, L. , Moretzsohn, M. C. , Shirasawa, K. , Huang, W. , Vidigal, B. , Abernathy, B. , Chu, Y. , Niederhuth, C. E. , Umale, P. , … Ozias‐Akins, P. (2016). The genome sequences of *Arachis duranensis* and *Arachis ipaensis*, the diploid ancestors of cultivated peanut. Nature Genetics, 48, 438–446. 10.1038/ng.3517 26901068

[pld3352-bib-0006] Bradbury, P. J. , Zhang, Z. , Kroon, D. E. , Casstevens, T. M. , Ramdoss, Y. , & Buckler, E. S. (2007). TASSEL: Software for association mapping of complex traits in diverse samples. Bioinformatics, 23, 2633–2635. 10.1093/bioinformatics/btm308 17586829

[pld3352-bib-0007] Bryant, J. A. , & Hughes, S. G. (2011). Vicia. In C. Kole (Ed.), Wild Crop Relatives: Genomic and Breeding Resources (pp. 273–289). Springer‐Verlag. 10.1007/978-3-642-14387-8_14

[pld3352-bib-0008] Cabanettes, F. , & Klopp, C. (2018). D‐GENIES: Dot plot large genomes in an interactive, efficient and simple way. PeerJ, 6, e4958. 10.7717/peerj.4958 29888139 PMC5991294

[pld3352-bib-0009] Cançado, G. (2011). The importance of genetic diversity to manage abiotic stress. In A. Shanker (Ed.), Abiotic stress in plants—Mechanisms and adaptations (pp. 351–366). InTech.

[pld3352-bib-0010] Cantarel, B. L. , Korf, I. , Robb, S. M. , Parra, G. , Ross, E. , Moore, B. , Holt, C. , Sanchez Alvarado, A. , & Yandell, M. (2008). MAKER: An easy‐to‐use annotation pipeline designed for emerging model organism genomes. Genome Research, 18, 188–196. 10.1101/gr.6743907 18025269 PMC2134774

[pld3352-bib-0011] Chan, P. P. , & Lowe, T. M. (2019). tRNAscan‐SE: Searching for tRNA Genes in Genomic Sequences. Methods in Molecular Biology, 1962, 1–14. 10.1007/978-1-4939-9173-0_1 31020551 PMC6768409

[pld3352-bib-0012] Cingolani, P. , Platts, A. , Wang, L. L. , Coon, M. , Nguyen, T. , Wang, L. , Land, S. J. , Lu, X. , & Ruden, D. M. (2012). A program for annotating and predicting the effects of single nucleotide polymorphisms, SnpEff: SNPs in the genome of *Drosophila melanogaster* strain w1118; iso‐2; iso‐3. Fly (Austin), 6, 80–92. 10.4161/fly.19695 22728672 PMC3679285

[pld3352-bib-0013] Danecek, P. , Auton, A. , Abecasis, G. , Albers, C. A. , Banks, E. , DePristo, M. A. , Handsaker, R. E. , Lunter, G. , Marth, G. T. , Sherry, S. T. , McVean, G. , Durbin, R. , & Genomes Project Analysis G . (2011). The variant call format and VCFtools. Bioinformatics, 27, 2156–2158. 21653522 10.1093/bioinformatics/btr330 PMC3137218

[pld3352-bib-0014] El‐Rodeny, W. , Kimura, M. , Hirakawa, H. , Sabah, A. , Shirasawa, K. , Sato, S. , Tabata, S. , Sasamoto, S. , Watanabe, A. , Kawashima, K. , Kato, M. , Wada, T. , Tsuruoka, H. , Takahashi, C. , Minami, C. , Nanri, K. , Nakayama, S. , Kohara, M. , Yamada, M. , … Isobe, S. (2014). Development of EST‐SSR markers and construction of a linkage map in faba bean (*Vicia faba*). Breeding Science, 64, 252–263. 10.1270/jsbbs.64.252 25320560 PMC4154614

[pld3352-bib-0015] English, A. C. , Richards, S. , Han, Y. , Wang, M. , Vee, V. , Qu, J. , Qin, X. , Muzny, D. M. , Reid, J. G. , Worley, K. C. , & Gibbs, R. A. (2012). Mind the gap: Upgrading genomes with Pacific Biosciences RS long‐read sequencing technology. PLoS ONE, 7, e47768. 10.1371/journal.pone.0047768 23185243 PMC3504050

[pld3352-bib-0016] Fernie, A. R. , & Yan, J. (2019). De novo domestication: An alternative route toward new crops for the future. Molecular Plant, 12, 615–631. 10.1016/j.molp.2019.03.016 30999078

[pld3352-bib-0017] Frazee, A. C. , Pertea, G. , Jaffe, A. E. , Langmead, B. , Salzberg, S. L. , & Leek, J. T. (2015). Ballgown bridges the gap between transcriptome assembly and expression analysis. Nature Biotechnology, 33, 243–246. 10.1038/nbt.3172 PMC4792117 25748911

[pld3352-bib-0018] Fu, Y. B. (2015). Understanding crop genetic diversity under modern plant breeding. Theoretical and Applied Genetics, 128, 2131–2142. 10.1007/s00122-015-2585-y 26246331 PMC4624815

[pld3352-bib-0019] Grabherr, M. G. , Haas, B. J. , Yassour, M. , Levin, J. Z. , Thompson, D. A. , Amit, I. , Adiconis, X. , Fan, L. , Raychowdhury, R. , Zeng, Q. , Chen, Z. , Mauceli, E. , Hacohen, N. , Gnirke, A. , Rhind, N. , di Palma, F. , Birren, B. W. , Nusbaum, C. , Lindblad‐Toh, K. , … Regev, A. (2011). Full‐length transcriptome assembly from RNA‐Seq data without a reference genome. Nature Biotechnology, 29, 644–652. 10.1038/nbt.1883 PMC3571712 21572440

[pld3352-bib-0020] Hackl, T. , Hedrich, R. , Schultz, J. , & Forster, F. (2014). proovread: Large‐scale high‐accuracy PacBio correction through iterative short read consensus. Bioinformatics, 30, 3004–3011. 10.1093/bioinformatics/btu392 25015988 PMC4609002

[pld3352-bib-0021] Hickey, L. T. , Hafeez, A. N. , Robinson, H. , Jackson, S. A. , Leal‐Bertioli, S. C. M. , Tester, M. , Gao, C. , Godwin, I. D. , Hayes, B. J. , & Wulff, B. B. H. (2019). Breeding crops to feed 10 billion. Nature Biotechnology, 37, 744–754. 10.1038/s41587-019-0152-9 31209375

[pld3352-bib-0022] Hill, P. , Burford, D. , Martin, D. M. , & Flavell, A. J. (2005). Retrotransposon populations of Vicia species with varying genome size. Molecular Genetics and Genomics, 273, 371–381. 10.1007/s00438-005-1141-x 15891910

[pld3352-bib-0023] Hoff, K. J. , Lange, S. , Lomsadze, A. , Borodovsky, M. , & Stanke, M. (2016). BRAKER1: Unsupervised RNA‐Seq‐based genome annotation with GeneMark‐ET and AUGUSTUS. Bioinformatics, 32, 767–769. 10.1093/bioinformatics/btv661 26559507 PMC6078167

[pld3352-bib-0024] Houben, A. (2017). B chromosomes—A matter of chromosome drive. Frontiers in Plant Science, 8, 210. 28261259 10.3389/fpls.2017.00210 PMC5309253

[pld3352-bib-0025] Izawa, T. , Konishi, S. , Shomura, A. , & Yano, M. (2009). DNA changes tell us about rice domestication. Current Opinion in Plant Biology, 12, 185–192. 10.1016/j.pbi.2009.01.004 19185529

[pld3352-bib-0026] Jones, P. , Binns, D. , Chang, H. Y. , Fraser, M. , Li, W. , McAnulla, C. , McWilliam, H. , Maslen, J. , Mitchell, A. , Nuka, G. , Pesseat, S. , Quinn, A. F. , Sangrador‐Vegas, A. , Scheremetjew, M. , Yong, S. Y. , Lopez, R. , & Hunter, S. (2014). InterProScan 5: Genome‐scale protein function classification. Bioinformatics, 30, 1236–1240. 10.1093/bioinformatics/btu031 24451626 PMC3998142

[pld3352-bib-0027] Kim, D. , Langmead, B. , & Salzberg, S. L. (2015). HISAT: A fast spliced aligner with low memory requirements. Nature Methods, 12, 357–360. 10.1038/nmeth.3317 25751142 PMC4655817

[pld3352-bib-0028] Kreplak, J. , Madoui, M. A. , Capal, P. , Novak, P. , Labadie, K. , Aubert, G. , Bayer, P. E. , Gali, K. K. , Syme, R. A. , Main, D. , Klein, A. , Bérard, A. , Vrbová, I. , Fournier, C. , d'Agata, L. , Belser, C. , Berrabah, W. , Toegelová, H. , Milec, Z. , … Burstin, J. (2019). A reference genome for pea provides insight into legume genome evolution. Nature Genetics, 51, 1411–1422. 10.1038/s41588-019-0480-1 31477930

[pld3352-bib-0029] Ladizinsky, G. (1998). Plant evolution under domestication. Kluwer Academic Publishers. 10.1007/978-94-011-4429-2

[pld3352-bib-0030] Ladizinsky, G. , & Waines, G. (1982). Seed protein polymorphism in *Vicia sativa* agg. (Fabaceae). Plant Systematics and Evolution, 141, 1–5. 10.1007/BF01006474

[pld3352-bib-0031] Langfelder, P. , & Horvath, S. (2008). WGCNA: An R package for weighted correlation network analysis. BMC Bioinformatics, 9, 559. 10.1186/1471-2105-9-559 19114008 PMC2631488

[pld3352-bib-0032] Langmead, B. , & Salzberg, S. L. (2012). Fast gapped‐read alignment with Bowtie 2. Nature Methods, 9, 357–359. 10.1038/nmeth.1923 22388286 PMC3322381

[pld3352-bib-0033] Li, H. , Handsaker, B. , Wysoker, A. , Fennell, T. , Ruan, J. , Homer, N. , Marth, G. , Abecasis, G. , Durbin, R. , & Genome Project Data Processing S . (2009). The sequence alignment/map format and SAMtools. Bioinformatics, 25, 2078–2079. 19505943 10.1093/bioinformatics/btp352 PMC2723002

[pld3352-bib-0034] Li, W. , & Godzik, A. (2006). Cd‐hit: A fast program for clustering and comparing large sets of protein or nucleotide sequences. Bioinformatics, 22, 1658–1659. 10.1093/bioinformatics/btl158 16731699

[pld3352-bib-0035] Link, W. , Ederer, W. , Metz, P. , Buiel, H. , & Melchinger, A. E. (1994). Genotypic and environmental variation for degree of cross‐fertilization in faba bean. Crop Science, 34, 960–964. 10.2135/cropsci1994.0011183X003400040024x

[pld3352-bib-0036] Litrico, I. , & Violle, C. (2015). Diversity in plant breeding: A new conceptual framework. Trends in Plant Science, 20, 604–613. 10.1016/j.tplants.2015.07.007 26440430

[pld3352-bib-0037] Luo, R. , Liu, B. , Xie, Y. , Li, Z. , Huang, W. , Yuan, J. , Guangzhu, H. , Chen, Y. , Pan, Q. , Liu, Y. , Tang, J. , Wu, G. , Zhang, H. , Shi, Y. , Liu, Y. , Yu, C. , Wang, B. , Lu, Y. , Han, C. , … Wang, J. (2012). SOAPdenovo2: An empirically improved memory‐efficient short‐read de novo assembler. Gigascience, 1, 18. 10.1186/2047-217X-1-18 23587118 PMC3626529

[pld3352-bib-0038] Macas, J. , Novak, P. , Pellicer, J. , Cizkova, J. , Koblizkova, A. , Neumann, P. , Fukova, I. , Dolezel, J. , Kelly, L. J. , & Leitch, I. J. (2015). In depth characterization of repetitive DNA in 23 plant genomes reveals sources of genome size variation in the legume tribe Fabeae. PLoS ONE, 10, e0143424. 10.1371/journal.pone.0143424 26606051 PMC4659654

[pld3352-bib-0039] Mammadov, J. , Buyyarapu, R. , Guttikonda, S. K. , Parliament, K. , Abdurakhmonov, I. Y. , & Kumpatla, S. P. (2018). Wild relatives of maize, rice, cotton, and soybean: Treasure troves for tolerance to biotic and abiotic stresses. Frontiers in Plant Science, 9, 886. 10.3389/fpls.2018.00886 30002665 PMC6032925

[pld3352-bib-0040] Marcais, G. , & Kingsford, C. (2011). A fast, lock‐free approach for efficient parallel counting of occurrences of k‐mers. Bioinformatics, 27, 764–770. 10.1093/bioinformatics/btr011 21217122 PMC3051319

[pld3352-bib-0041] Mendez‐Vigo, B. , Pico, F. X. , Ramiro, M. , Martinez‐Zapater, J. M. , & Alonso‐Blanco, C. (2011). Altitudinal and climatic adaptation is mediated by flowering traits and FRI, FLC, and PHYC genes in Arabidopsis. Plant Physiology, 157, 1942–1955. 10.1104/pp.111.183426 21988878 PMC3327218

[pld3352-bib-0042] Mundt, C. C. (2002). Use of multiline cultivars and cultivar mixtures for disease management. Annual Review of Phytopathology, 40, 381–410. 10.1146/annurev.phyto.40.011402.113723 12147765

[pld3352-bib-0043] Navratilova, A. , Neumann, P. , & Macas, J. (2003). Karyotype analysis of four Vicia species using in situ hybridization with repetitive sequences. Annals of Botany, 91, 921–926. 10.1093/aob/mcg099 12770847 PMC4242401

[pld3352-bib-0044] Nouzova, M. , Neumann, P. , Navratilova, A. , Galbraith, D. W. , & Macas, J. (2001). Microarray‐based survey of repetitive genomic sequences in Vicia spp. Plant Molecular Biology, 45, 229–244. 10.1023/A:1006408119740 11289513

[pld3352-bib-0045] Ogata, H. , Goto, S. , Sato, K. , Fujibuchi, W. , Bono, H. , & Kanehisa, M. (1999). KEGG: Kyoto encyclopedia of genes and genomes. Nucleic Acids Research, 27, 29–34. 10.1093/nar/27.1.29 9847135 PMC148090

[pld3352-bib-0046] Pearce, S. R. , Harrison, G. , Li, D. , Heslop‐Harrison, J. , Kumar, A. , & Flavell, A. J. (1996). The Ty1‐copia group retrotransposons in Vicia species: Copy number, sequence heterogeneity and chromosomal localisation. Molecular & General Genetics, 250, 305–315. 10.1007/BF02174388 8602145

[pld3352-bib-0047] Pertea, M. , Kim, D. , Pertea, G. M. , Leek, J. T. , & Salzberg, S. L. (2016). Transcript‐level expression analysis of RNA‐seq experiments with HISAT, StringTie and Ballgown. Nature Protocols, 11, 1650–1667. 10.1038/nprot.2016.095 27560171 PMC5032908

[pld3352-bib-0048] Pertea, M. , Pertea, G. M. , Antonescu, C. M. , Chang, T. C. , Mendell, J. T. , & Salzberg, S. L. (2015). StringTie enables improved reconstruction of a transcriptome from RNA‐seq reads. Nature Biotechnology, 33, 290–295. 10.1038/nbt.3122 PMC4643835 25690850

[pld3352-bib-0049] Peterson, B. K. , Weber, J. N. , Kay, E. H. , Fisher, H. S. , & Hoekstra, H. E. (2012). Double digest RADseq: An inexpensive method for de novo SNP discovery and genotyping in model and non‐model species. PLoS ONE, 7, e37135. 10.1371/journal.pone.0037135 22675423 PMC3365034

[pld3352-bib-0050] Price, A. L. , Jones, N. C. , & Pevzner, P. A. (2005). De novo identification of repeat families in large genomes. Bioinformatics, 21(Suppl 1), i351–i358. 10.1093/bioinformatics/bti1018 15961478

[pld3352-bib-0051] Sato, S. , Nakamura, Y. , Kaneko, T. , Asamizu, E. , Kato, T. , Nakao, M. , Sasamoto, S. , Watanabe, A. , Ono, A. , Kawashima, K. , Fujishiro, T. , Katoh, M. , Kohara, M. , Kishida, Y. , Minami, C. , Nakayama, S. , Nakazaki, N. , Shimizu, Y. , Shinpo, S. , … Tabata, S. (2008). Genome structure of the legume, *Lotus japonicus* . DNA Research, 15, 227–239. 10.1093/dnares/dsn008 18511435 PMC2575887

[pld3352-bib-0052] Schmieder, R. , & Edwards, R. (2011). Quality control and preprocessing of metagenomic datasets. Bioinformatics, 27, 863–864. 10.1093/bioinformatics/btr026 21278185 PMC3051327

[pld3352-bib-0053] Schmutz, J. , McClean, P. E. , Mamidi, S. , Wu, G. A. , Cannon, S. B. , Grimwood, J. , Jenkins, J. , Shu, S. , Song, Q. , Chavarro, C. , Torres‐Torres, M. , Geffroy, V. , Moghaddam, S. M. , Gao, D. , Abernathy, B. , Barry, K. , Blair, M. , Brick, M. A. , Chovatia, M. , … Jackson, S. A. (2014). A reference genome for common bean and genome‐wide analysis of dual domestications. Nature Genetics, 46, 707–713. 10.1038/ng.3008 24908249 PMC7048698

[pld3352-bib-0054] Schneider, C. A. , Rasband, W. S. , & Eliceiri, K. W. (2012). NIH Image to ImageJ: 25 years of image analysis. Nature Methods, 9, 671–675. 10.1038/nmeth.2089 22930834 PMC5554542

[pld3352-bib-0055] Shirasawa, K. , Hirakawa, H. , & Isobe, S. (2016). Analytical workflow of double‐digest restriction site‐associated DNA sequencing based on empirical and in silico optimization in tomato. DNA Research, 23, 145–153. 10.1093/dnares/dsw004 26932983 PMC4833422

[pld3352-bib-0056] Simao, F. A. , Waterhouse, R. M. , Ioannidis, P. , Kriventseva, E. V. , & Zdobnov, E. M. (2015). BUSCO: Assessing genome assembly and annotation completeness with single‐copy orthologs. Bioinformatics, 31, 3210–3212. 10.1093/bioinformatics/btv351 26059717

[pld3352-bib-0057] Tatusov, R. L. , Fedorova, N. D. , Jackson, J. D. , Jacobs, A. R. , Kiryutin, B. , Koonin, E. V. , Krylov, D. M. , Mazumder, R. , Mekhedov, S. L. , Nikolskaya, A. N. , Rao, B. S. , Smirnov, S. , Sverdlov, A. V. , Vasudevan, S. , Wolf, Y. I. , Yin, J. J. , & Natale, D. A. (2003). The COG database: An updated version includes eukaryotes. BMC Bioinformatics, 4, 41. 10.1186/1471-2105-4-41 12969510 PMC222959

[pld3352-bib-0058] Vaughan, D. A. , Balazs, E. , & Heslop‐Harrison, J. S. (2007). From crop domestication to super‐domestication. Annals of Botany, 100, 893–901. 10.1093/aob/mcm224 17940074 PMC2759215

[pld3352-bib-0059] Young, N. D. , Debelle, F. , Oldroyd, G. E. , Geurts, R. , Cannon, S. B. , Udvardi, M. K. , Benedito, V. A. , Mayer, K. F. X. , Gouzy, J. , Schoof, H. , Van de Peer, Y. , Proost, S. , Cook, D. R. , Meyers, B. C. , Spannagl, M. , Cheung, F. , De Mita, S. , Krishnakumar, V. , Gundlach, H. , … Roe, B. A. (2011). The Medicago genome provides insight into the evolution of rhizobial symbioses. Nature, 480, 520–524. 10.1038/nature10625 22089132 PMC3272368

[pld3352-bib-0060] Zhu, Y. , Chen, H. , Fan, J. , Wang, Y. , Li, Y. , Chen, J. , Fan, J. , Yang, S. , Hu, L. , Leung, H. , Mew, T. W. , Teng, P. S. , Wang, Z. , & Mundt, C. C. (2000). Genetic diversity and disease control in rice. Nature, 406, 718–722. 10.1038/35021046 10963595

