# Supplementary material for: Genome features of common vetch ( Vicia sativa ) in natural habitats
Source: Plant Direct. 2021 Oct 7;5(10):e352. doi: 10.1002/pld3.352 (PMC8496506; doi:10.1002/pld3.352)
Supplement: Supplementary file 2 — Figure S1 Cross‐validation errors for 12 natural populations of Vicia sativa from Japan in admixture analysis. Figure S2 Nucleotide diversity of SNP modules across 12 natural populations of V. sativa from Japan. [file PLD3-5-e352-s001.pdf]

Genome features of common vetch (*Vicia sativa*) in natural habitats

Kenta Shirasawa[1*], Shunichi Kosugi[1†], Kazuhiro Sasaki[2‡], Andrea Ghelfi[1], Koei Okazaki[1], Atsushi Toyoda[3], Hideki Hirakawa[1], Sachiko Isobe[1]

[1]Kazusa DNA Research Institute, Kisarazu, Chiba 292-0818, Japan, [2]Institute for Sustainable Agro-ecosystem Services, Graduate School of Agricultural and Life Sciences, The University of Tokyo, Nishitokyo, Tokyo 188-0001, Japan, and [3]National Institute of Genetics, Mishima, Shizuoka 411-8540, Japan

[†] Present address: RIKEN, Yokohama, Kanagawa 230-0045, Japan

[‡] Present address: Japan International Research Center for Agricultural Sciences, Tsukuba, Ibaraki 305-8686, Japan

*Corresponding author: Kenta Shirasawa (shirasaw@kazusa.or.jp)

Tel. +81-438 52 3935

Supplementary Table S1 Plant materials

Supplementary Table S2 Genome and transcriptome data

Supplementary Table S3 Number of KOG functions for protein-encoding genes

Supplementary Table S4 Number of genes mapped to KEGG pathways

Supplementary Table S5 Number of GO terms for protein-encoding genes

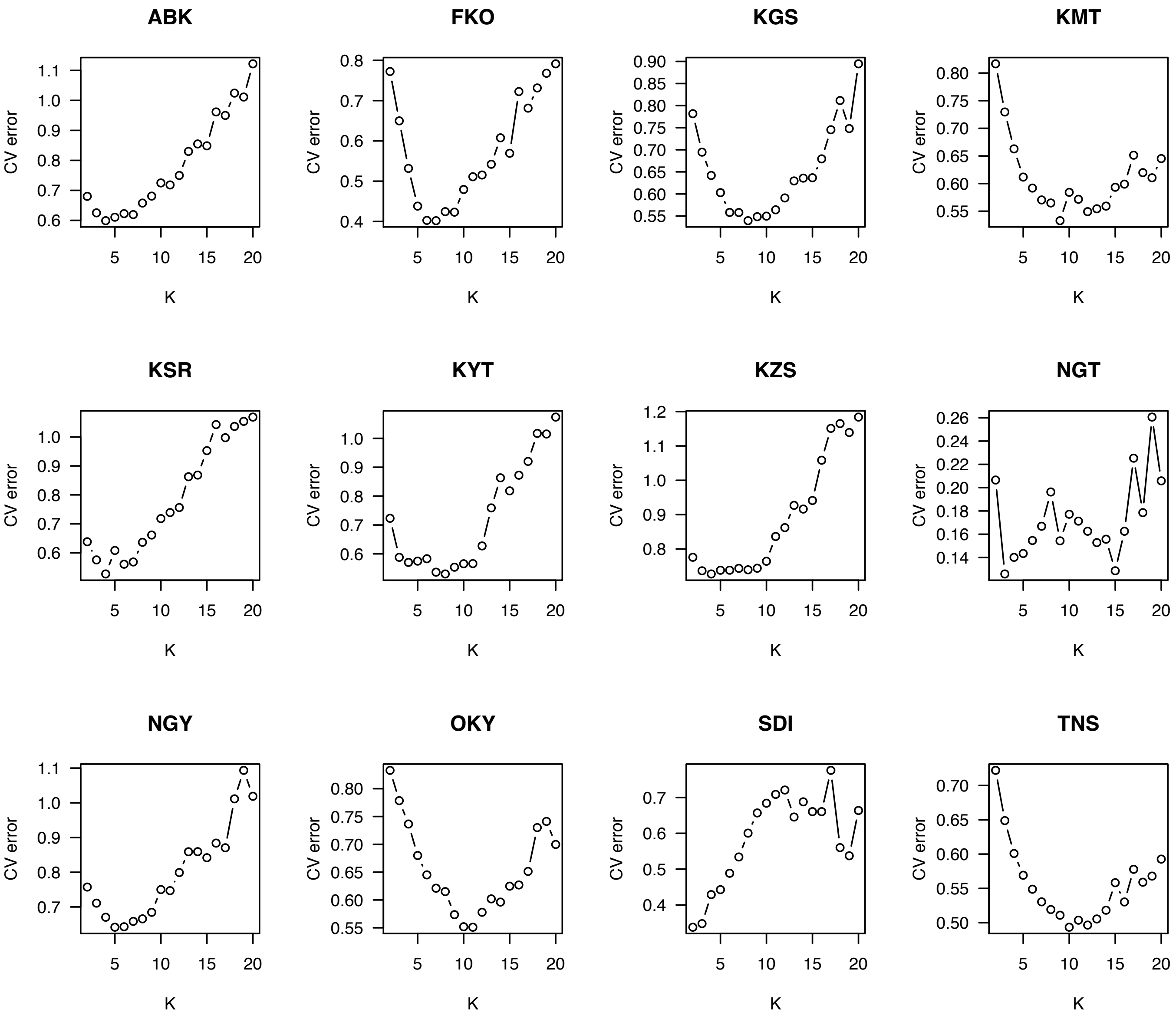

Supplementary Figure S1 Cross-validation errors for 12 natural populations of *Vicia sativa* from Japan in admixture analysis.

Three-letter codes indicate sampling location in Japan as shown in Supplementary Table S1 and Supplementary Figure S1.

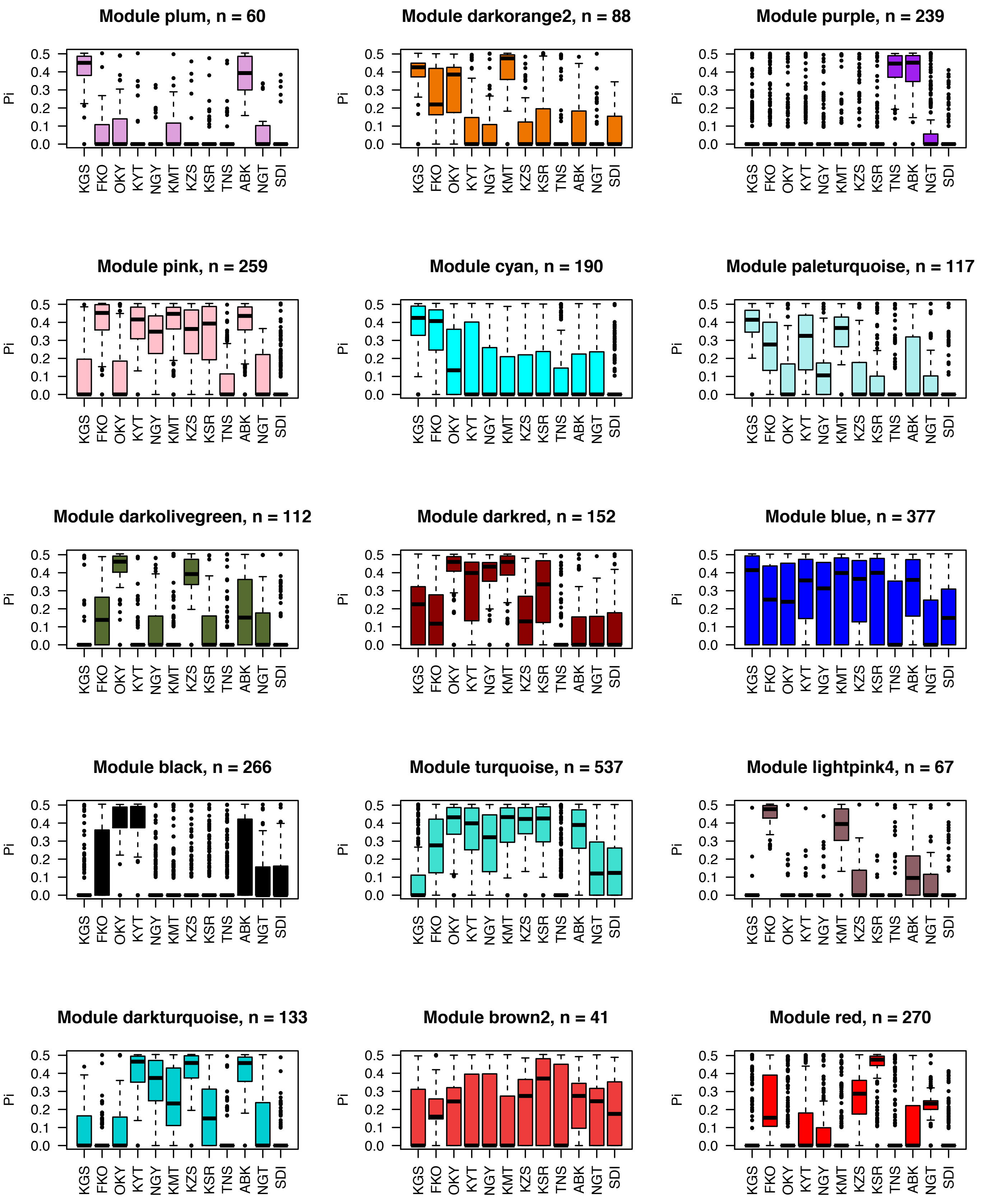

Supplementary Figure S2 Nucleotide diversity of SNP modules across 12 natural populations of *Vicia sativa* from Japan.

Numbers of SNPs in each module are shown at the tops of boxplots. Three-letter codes indicate sampling location in Japan as shown in Supplementary Table S1 and Supplementary Figure S1.

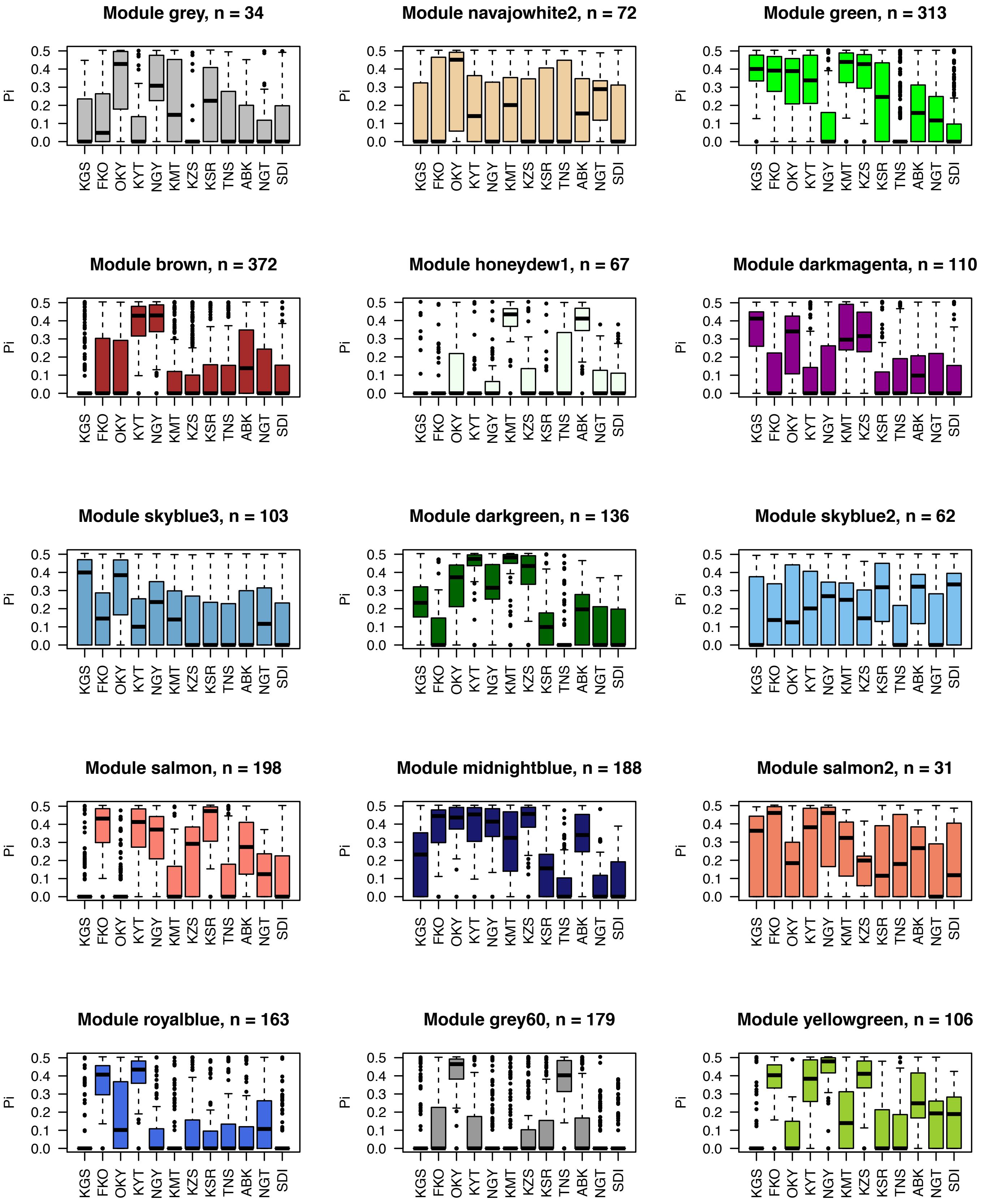

Supplementary Figure S2 (continued)

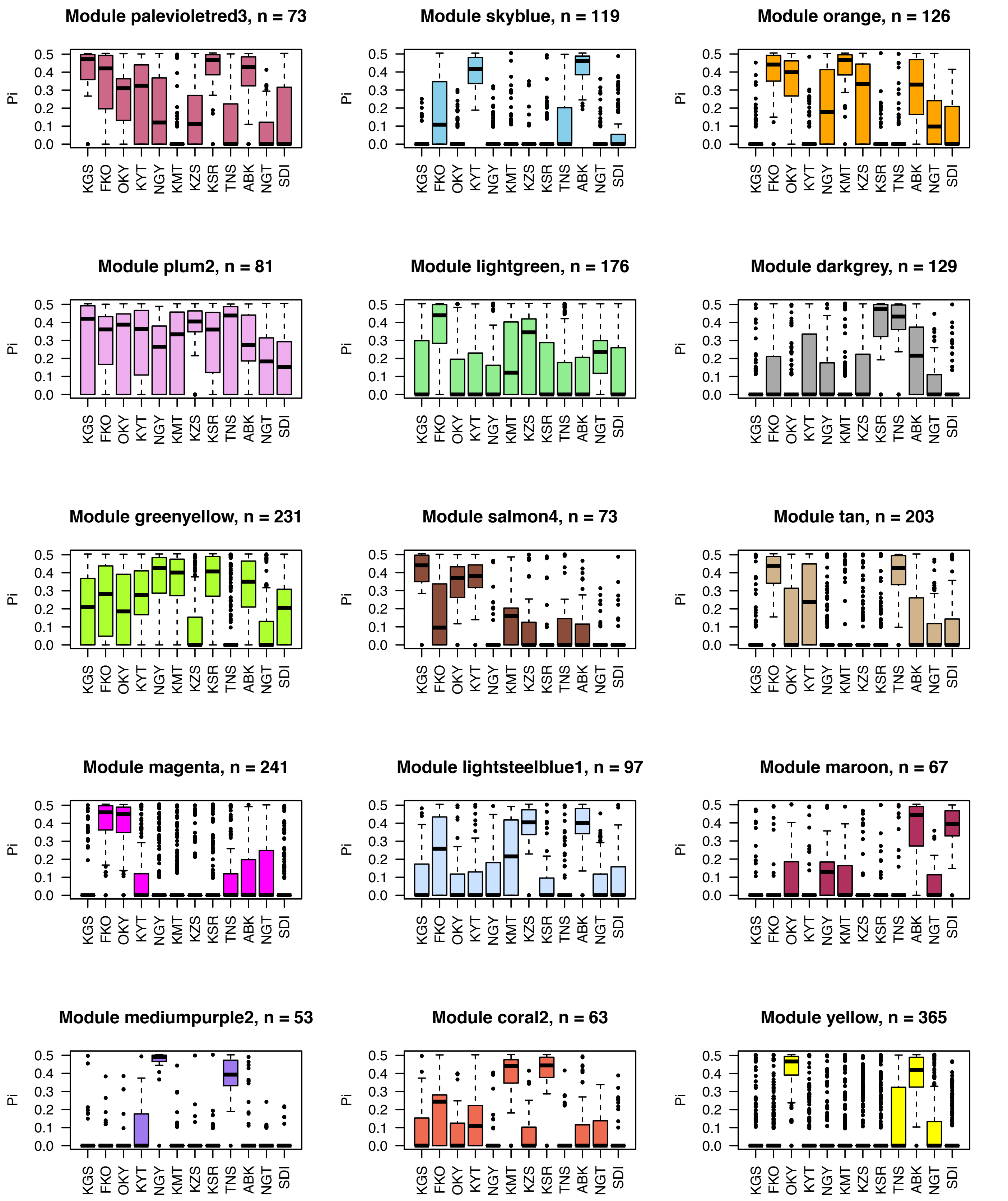

Supplementary Figure S2 (continued)

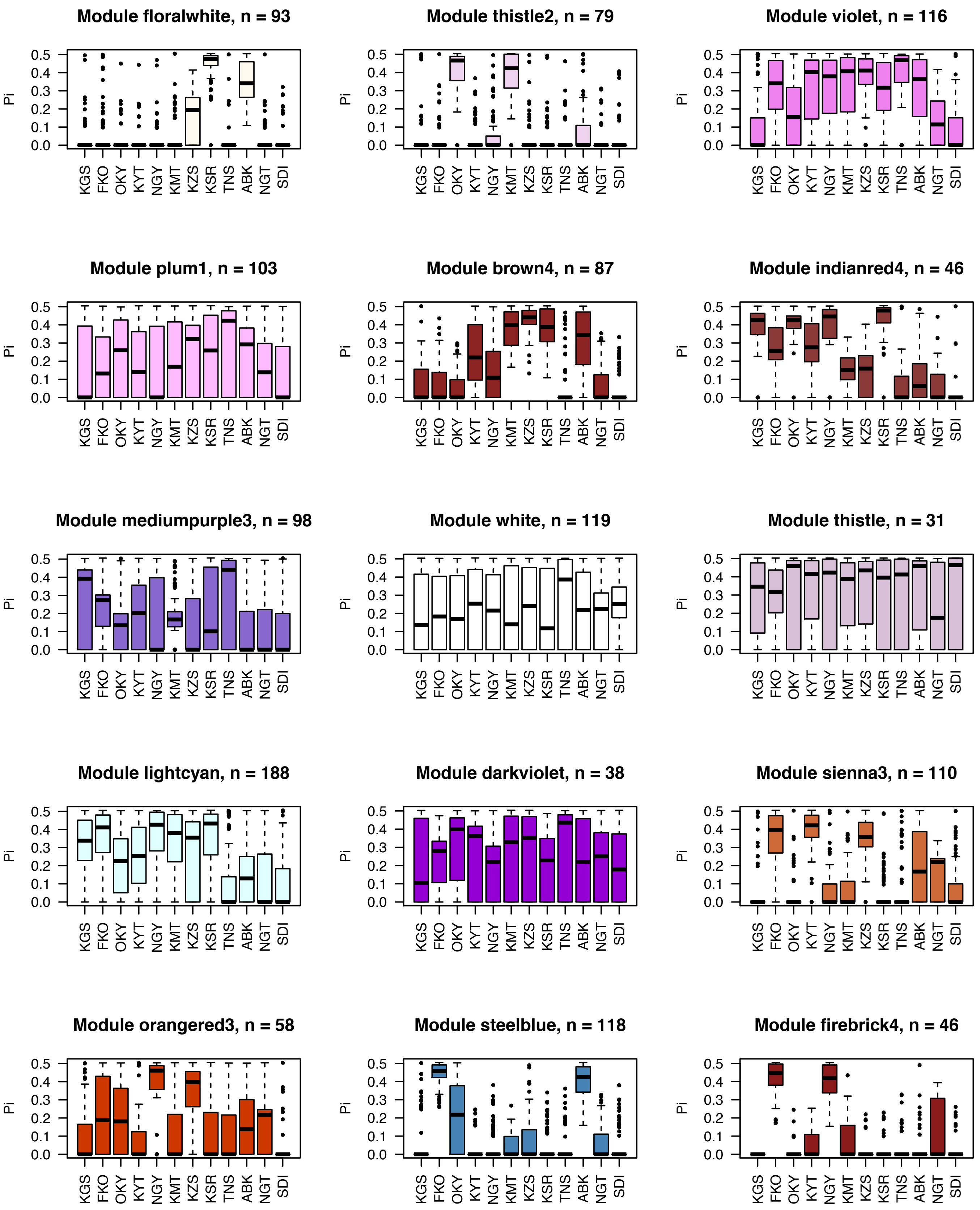

Supplementary Figure S2 (continued)

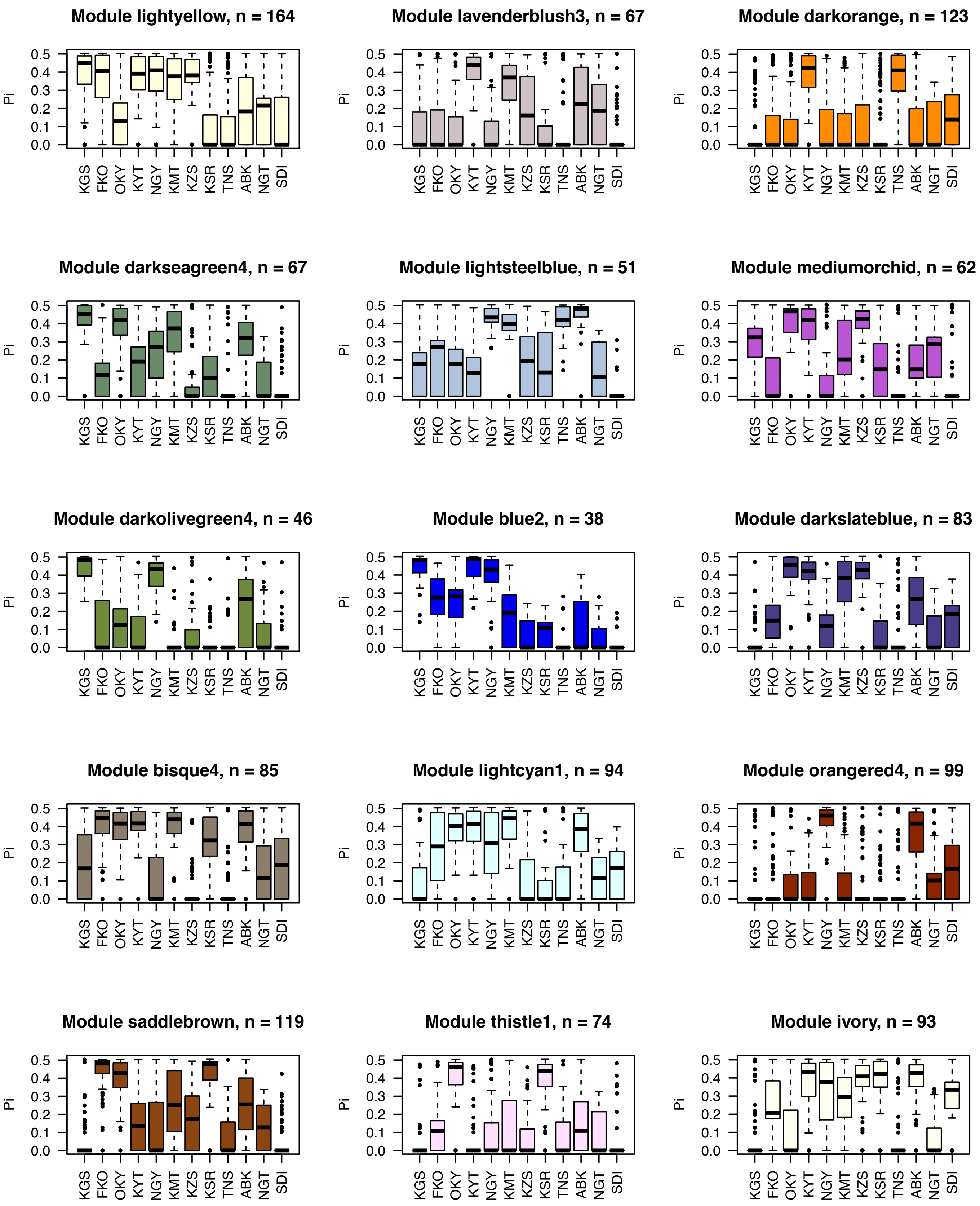

Supplementary Figure S2 (continued)

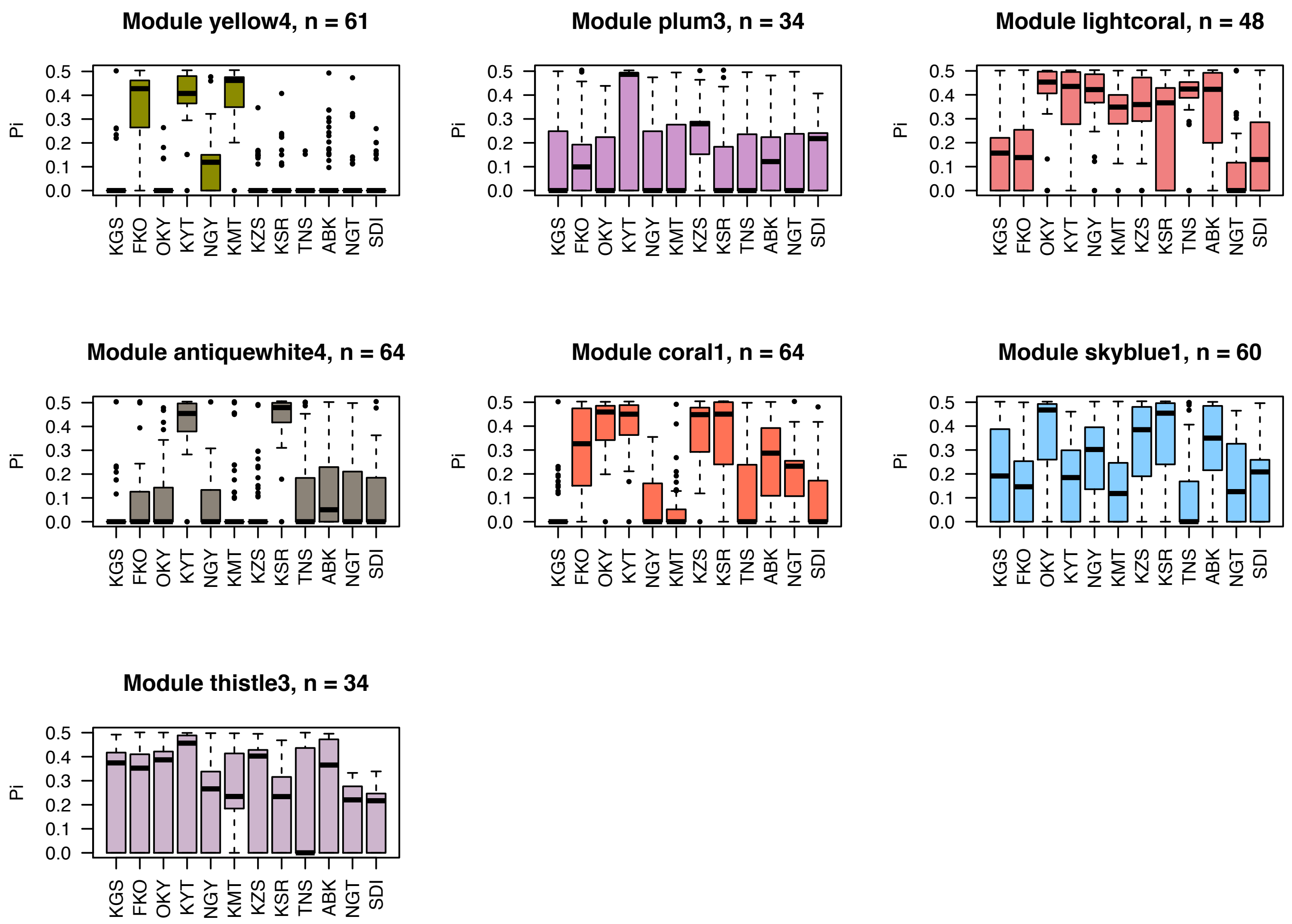

Supplementary Figure S2 (continued)
